# Sensing infection and tissue damage

**DOI:** 10.15252/emmm.201607227

**Published:** 2017-01-24

**Authors:** Caetano Reis e Sousa

**Affiliations:** ^1^ Immunobiology LaboratoryThe Francis Crick InstituteLondonUK

**Keywords:** Immunology

## Abstract

Innate and adaptive immunity work concertedly in vertebrates to restore homoeostasis following pathogen invasion or other insults. Like all homoeostatic circuits, immunity relies on an integrated system of sensors, transducers and effectors that can be analysed in cellular or molecular terms. At the cellular level, T and B lymphocytes act as an effector arm of immunity that is mobilised in response to signals transduced by innate immune cells that detect a given insult. These innate cells are spread around the body and include dendritic cells (DCs), the chief immune sensors of pathogen invasion and tumour growth. At the molecular level, DCs possess receptors that directly sense pathogen presence and tissue damage and that signal via transduction pathways to control antigen presentation or regulate a plethora of genes encoding effector proteins that regulate immunity. Notably, molecular circuits for pathogen detection are not confined to DCs or even to immune cells. All cells express sensors and transducers that monitor invasion by viruses and bacteria and elicit suitable effector barriers to pathogen propagation. Here, I discuss work from my laboratory that has contributed to our understanding of these issues over the years.

## Cell‐intrinsic immunity to RNA viruses

**Figure 1:**
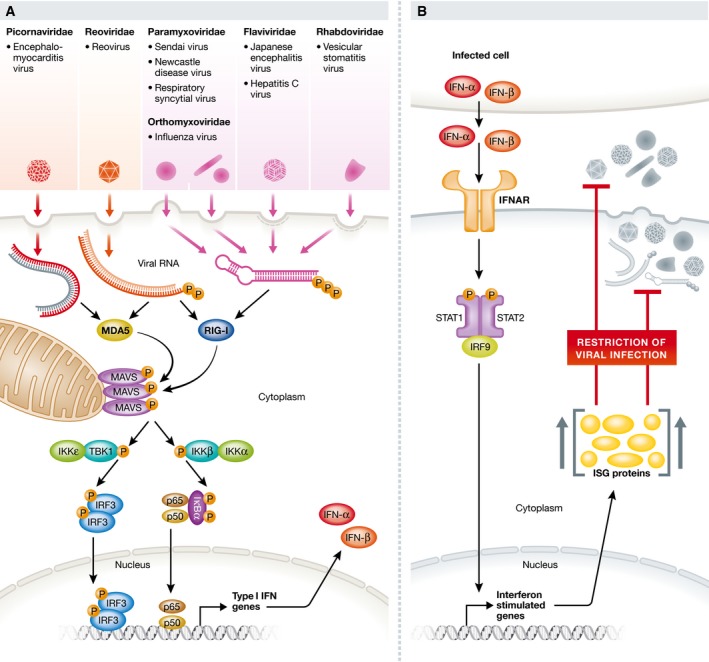
The IFN pathway of cell‐intrinsic antiviral immunity (A, B) Different viruses possess or generate RNAs that can be detected by RIG‐I and/or MDA5. These sensors transduce signals via the mitochondria‐associated MAVS protein that oligomerises and activates TBK‐1 and IKKα, IKKβ and IKKε to phosphorylate IRF transcription factor family members (e.g. IRF3 as depicted), as well as IκB to release NF‐κB family transcription factors p60 and p65. These transcription factors co‐ordinately induce expression of type I and type III IFN and other genes. IFNs are secreted and type I IFNs (IFNα/β) act in autocrine or paracrine manner via the type I IFN receptor (IFNAR) and STAT1/STA2/IRF9 signalling to induce expression of ISGs, the products of which restrict virus infection. P, phosphate group.

The ability of all nucleated vertebrate cells to respond to virus invasion has been recognised since the discovery of interferons, virus‐induced cytokines produced by both immune and non‐immune cells. However, only recently have the relevant sensors and transducers been identified. RNA viruses such as influenza virus replicate using a primer‐independent mechanism that leaves a tri‐phosphorylated nucleotide at the 5′ end of the genome. The 5′ppp mark is absent from cellular RNAs, which are capped (mRNA) or otherwise processed (rRNA and tRNA). 5′ppp and, for some viruses, 5′pp, therefore acts as a tell‐tale sign of RNA virus presence in the cytosol that is recognised, together with elements of RNA secondary structure, by a cytoplasmic protein sensor named RIG‐I (Pichlmair *et al*, 2006; Goubau *et al*, 2014). A related sensor, MDA5, recognises double stranded (ds) or highly base‐paired RNA, which is also often a product of viral replication and is absent from uninfected cells. Following activation by viral RNA, RIG‐I and MDA5 engage the mitochondrial adaptor protein MAVS initiating a signal transduction pathway that culminates in activation of transcription factors of the IRF and NF‐κB families to induce type I and type III IFN gene expression (Fig 1). Interestingly, IFNs are not themselves antiviral effectors. Rather, they are secreted by virally infected cells and act in an autocrine and paracrine amplification loop, binding to IFN receptors that signal to induce interferon‐stimulated genes (ISGs; Fig 1). ISGs include the RIG‐I and MDA5 sensors themselves, providing a positive feedback loop for innate virus sensing. ISGs also encode a variety of effector proteins that restrict virus propagation by shutting down cell translation, cleaving cellular and viral RNAs and blocking virion replication, assembly and/or release. As such, ISGs encode effectors of antiviral immunity elicited by a simple cell‐intrinsic sensing and transducing immune circuit, albeit one involving IFN‐mediated amplification and spread (Fig 1).

Interestingly, the IFN response is absent in invertebrates and plants, which, instead, defend themselves from viruses using RNA interference (RNAi). In those organisms, Dicer enzymes process viral dsRNA to generate small interfering RNA (siRNA) that is loaded onto a complex containing Argonaute proteins that can “slice” viral RNAs bearing complementary sequences. This sequence‐specific antiviral RNAi response was thought to have been lost during vertebrate evolution of the IFN response even though the RNAi machinery itself has been preserved and is used for miRNAs generation and action. In fact, sequence‐specific antiviral RNAi is not absent in mammals and was recently found to be masked or suppressed by the sequence‐unspecific actions of ISG proteins (Maillard *et al*, 2016). Therefore, RNAi is an antiviral strategy that is preserved from plants to humans and may be important in cellular niches in which the IFN response is attenuated.

## Dendritic cells as sensors of viruses and microbes

**Figure 2:**
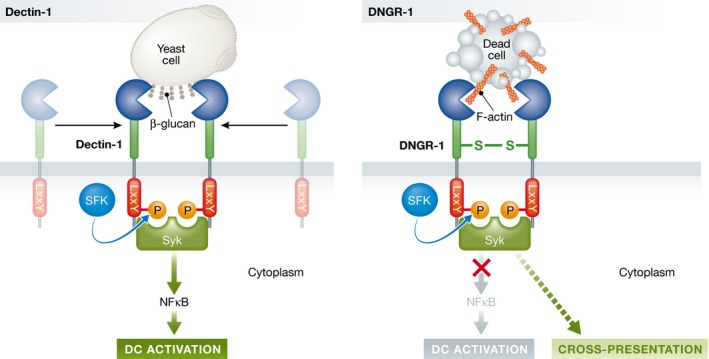
Role of the Syk‐coupled CLRs, Dectin‐1 and DNGR‐1, in DCs Dectin‐1 and DNGR‐1 can both be expressed by DCs. Dectin‐1 is monomeric but can oligomerise upon binding to β‐glucans exposed by fungal cells. Src family kinases (SFKs) phosphorylate the tyrosine in the YxxL hemITAM motif and allow for docking and activation of Syk, which then signals to NF‐κB, MAPK and NFAT resulting in DC activation. In contrast, DNGR‐1 is a homodimer stabilised by a disulphide bond in its neck region. Binding of DNGR‐1 to F‐actin on dead cells also leads to SFK‐dependent hemITAM phosphorylation and Syk activation, but this does not transmit to NF‐κB and does not induce DC activation. Rather, DNGR‐1 signalling appears to regulate endosomal maturation to favour presentation of dead cell‐associated antigens by MHC class I molecules, a process known as cross‐presentation.

Viruses have evolved measures to block cell‐intrinsic immunity; in vertebrates, innate defences are not sufficient to prevent their spread. We have a sophisticated system of adaptive immune defence that makes use of T and B cells that specifically recognise viruses and other pathogens, as well as commensals, at any body barrier that might be colonised. The T and B cells need to be primed by virus‐sensing DCs and RNA viruses present in extracellular spaces can be detected by specialised plasmacytoid DCs using transmembrane receptors of the Toll‐like receptor (TLR) family such as TLR7 (Diebold *et al*, 2004). The ligand‐binding domain of TLR7 faces the lumen of endosomes and detects the presence of RNA carried by influenza and other RNA viruses that are taken up into those compartments prior to low pH‐induced fusion and cytoplasmic entry (Diebold *et al*, 2004). TLR7 signalling in plasmacytoid DCs results, among others, in the production of type I IFNs that favour priming of antiviral effector T cells. Interestingly, TLR7 recognition, unlike that of RIG‐I, does not rely on virus‐specific RNA marks and the receptor can be triggered by self RNA artificially delivered to endosomal compartments (Diebold *et al*, 2004). This argues that pathogen detection can ensue, in some instances, from recognition of molecules shared between invader and host but that become mislocalised in an infectious setting (Diebold *et al*, 2004). Nevertheless, many TLRs and other innate immune sensing receptors specifically detect molecular signatures of microbes that are qualitatively distinct from those of self. Such receptors include Dectin‐1, a transmembrane protein member of the C‐type lectin receptor (CLR) family that binds to fungal and bacterial β‐1,3‐glucans in the extracellular space and endosomes. Dectin‐1 possesses a tyrosine‐based hemITAM signalling motif in its cytoplasmic tail that becomes phosphorylated by Src family kinases after ligand engagement and serves as a platform for recruiting Syk, a non‐receptor tyrosine kinase (Rogers *et al*, 2005). This initiates a cascade that results in triggering of NF‐κB, MAPK and NFAT signalling modules (Fig 2). DCs activated by Dectin‐1 signalling are competent to prime T cells, favouring the induction of Th17 cells, a CD4^+^ effector T‐cell type that mediates immunity to fungi and extracellular bacteria (LeibundGut‐Landmann *et al*, 2007).

## Detecting damage

Our gut, skin and other barrier surfaces are home to 1–10 times as many bacteria and fungi as our total content of human cells. Yet we do not react to commensals like we do to pathogens despite the fact that they all share the microbial signatures recognised by many innate immune receptors. It is still unclear how our immune system discriminates pathogens from commensals, but it is interesting to consider that tissue invasion and disruption are hallmarks of pathogenicity, as they are of malignancy. Interestingly, innate immune cells have receptors that allow detection of cytoplasmic and nuclear components that are exposed or released by damaged cells that have undergone cytopathic insult. One of these, DNGR‐1 (a.k.a. CLEC9A), is expressed selectively by DCs and binds to cells that have lost plasma membrane integrity (Sancho *et al*, 2009). Like Dectin‐1, DNGR‐1 is a hemITAM‐bearing transmembrane CLR that samples the extracellular and endosomal space and signals via Src and Syk (Sancho *et al*, 2009). Yet DNGR‐1 signalling in response to dead cell encounter does not couple to downstream activation of NF‐κB (Fig 2). Rather, it modulates endosomal maturation and has a specialised role in permitting DCs to extract antigens from cell corpses and present them to CD8^+^ T cells (Sancho *et al*, 2009). DNGR‐1‐dependent detection of dead cells plays a role in CD8^+^ T‐cell responses to cytopathic viruses and, likely, to cancer, in which cell death induced by hypoxia and/or therapy is an important determinant of immunity.

The ability of DNGR‐1 to detect dead cells is due to the fact that it binds to F‐actin, which is exposed when cells lose plasma membrane integrity (Ahrens *et al*, 2012). In retrospect, it makes ample sense that exposure of actin, a highly abundant and ubiquitous cytoskeletal protein, should be a target for innate detection of damaged cells. Notably, actin is so conserved that human or mouse DNGR‐1 can bind to F‐actin from all tested species, from yeast to humans (Ahrens *et al*, 2012). One might therefore anticipate that extracellular actin sensing preceded the evolution of adaptive immunity and constitutes an ancient mechanism for detecting tissue injury, with its roots perhaps in tissue repair. Consistent with that notion, actin injection into *Drosophila melanogaster* induces sterile JAK/STAT responses akin to ones previously seen in the context of injury or stress (Srinivasan *et al*, 2016). Interestingly, although there is no DNGR‐1 in *Drosophila*, the response requires the fly orthologues of Src and Syk arguing for possible evolutionary conservation of the pathway of extracellular actin detection (Srinivasan *et al*, 2016).

## Dendritic cell variety

DCs are part of a broader family of phagocytes that includes monocytes, macrophages and granulocytes. In fact, it is often hard to distinguish DCs from monocytes and macrophages leading to debate over their identity and function. Adding to the confusion, DCs are not one cell type but a family of cells that display similarities but also distinct molecular signatures and ontogenetic dependencies. In the mouse, DNGR‐1 is a useful marker of hematopoietic cell commitment to the DC lineage, being first expressed at low levels in DC precursors before they leave the bone marrow and colonise all tissues to give rise to the network of sentinel DCs (Schraml *et al*, 2013). Therefore, DNGR‐1‐mediated fate mapping is a powerful means of genetically tracing the DC lineage *in vivo* (Schraml *et al*, 2013). However, the receptor is also expressed at much higher levels by a particular sub‐type of fully differentiated DCs known as DC1 that is key for inducing antiviral and antitumour CD8^+^ T‐cell responses. Interestingly, pre‐clinical studies suggest that targeting antigens to DC1 via antibodies to DNGR‐1 is a promising approach for inducing or boosting antitumour immunity. Therefore, receptors utilised by DCs to sense cell damage or pathogens can be useful targets for manipulating the cells in the context of vaccination or immunotherapy. Thus, study of the mechanisms utilised by the immune system to detect pathogens and cell damage not only can lead to basic discoveries but can also have translational application.

## Conflict of interest

The author declares that he has no conflict of interest.
